# Assessing the Acceptability of a Tenofovir-Based Rectal Microbicide Douche among Young Men who Have Sex with Men: Results from ATN-DREAM (ATN 163)

**DOI:** 10.1007/s10461-025-04763-2

**Published:** 2025-05-29

**Authors:** José A. Bauermeister, Willey Lin, Jessica Webster, Louis L. Listerud, Tyler Burgese, Allison Agwu, Jessica Coleman Lewis, Thuy Anderson, Lisa Hightow-Weidman, Craig Hendrix, Renata Arrington-Sanders

**Affiliations:** 1 University of Pennsylvania, Philadelphia, PA USA; 2 Johns Hopkins University, Baltimore, MD USA; 3 Florida State University, Tallahassee, FL USA; 4 Children’s Hospital of Philadelphia, Philadelphia, PA USA

**Keywords:** Pre-exposure prophylaxis (PrEP), Prevention, Receptive anal intercourse, Adolescent health

## Abstract

Young men who have sex with men (YMSM) remain disproportionately affected by HIV, yet adherence to daily oral pre-exposure prophylaxis (PrEP) remains suboptimal. Rectal microbicides formulated as douches offer a behaviorally congruent alternative by leveraging existing douching practices. This study assessed the acceptability of a tenofovir-based rectal microbicide douche among YMSM. Eight YMSM (M = 20.9 years; range 18–24 years) participated in a Phase I, open-label, single-arm trial evaluating the safety, pharmacokinetics, and tolerability of a single 600 mg dose of tenofovir delivered in a 125mL rectal douche (NCT04686279). This mixed-methods study included post-dosing behavioral surveys and qualitative in-depth interviews (IDIs) to assess acceptability, tolerability, and perceptions of the product’s usability. Interviews were transcribed, coded, and analyzed thematically. Descriptive statistics summarized participant characteristics, douching behaviors, and willingness to use the product in different contexts. Participants reported high acceptability of the rectal microbicide douche, particularly its ease of use and integration into existing sexual health routines. Most (87.5%) indicated they would find the product acceptable for HIV prevention, and 75% reported they would likely use it before every receptive anal intercourse (RAI) event. The episodic dosing regimen was perceived as a benefit, especially for casual sexual encounters. Barriers included product design concerns, privacy, and cost. A rectal microbicide douche was highly acceptable among YMSM, supporting its potential as a novel HIV prevention strategy. Findings underscore the importance of behaviorally congruent interventions and highlight key considerations for future product development, including improved packaging, affordability, and accessibility.

Trials Registration: NCT04686279.

## Resumen

Los hombres jóvenes que tienen sexo con hombres (YMSM) continúan siendo desproporcionadamente afectados por el VIH, y la adherencia a la PrEP oral diaria sigue siendo baja. Los microbicidas rectales en forma de duchas ofrecen una alternativa conductualmente congruente al aprovechar prácticas de higiene rectal ya existentes. Este estudio evaluó la aceptabilidad de una ducha rectal con tenofovir entre YMSM. Ocho participantes (edad media = 20.9 años; rango: 18–24) participaron en un ensayo fase I, abierto y de un solo brazo, que evaluó la seguridad, farmacocinética y tolerabilidad de una dosis única de 600 mg de tenofovir en una ducha rectal de 125mL (NCT04686279). Se utilizó un enfoque de métodos mixtos que incluyó encuestas posteriores a la dosificación y entrevistas cualitativas en profundidad. Las entrevistas se analizaron temáticamente y se usaron estadísticas descriptivas para resumir características de los participantes y comportamientos de uso. Los participantes reportaron alta aceptabilidad, especialmente por su facilidad de uso e integración en sus rutinas sexuales. El 87.5% consideró aceptable el producto para prevenir el VIH, y el 75% indicó que probablemente lo usaría antes de cada encuentro de sexo anal receptivo. El régimen de dosificación episódico fue percibido como una ventaja para encuentros sexuales casuales. Las principales barreras fueron el diseño del producto, preocupaciones de privacidad y el costo. Los resultados apoyan el potencial de esta ducha rectal como una estrategia novedosa de prevención del VIH y resaltan la necesidad de intervenciones adaptadas a los comportamientos reales, con mejoras en empaque, accesibilidad y asequibilidad.

## Introduction

Young men who have sex with men (YMSM) remain disproportionately affected by the HIV epidemic in the United States (U.S.). Despite the availability of highly effective biomedical prevention strategies, including daily oral pre-exposure prophylaxis (PrEP), uptake and adherence among YMSM remain suboptimal due to structural barriers, stigma, and individual-level adherence challenges [1–6]​. As a result, there is growing interest in developing behaviorally congruent HIV prevention modalities that align with YMSM’s existing sexual health practices [2, 6–8]​. One such strategy involves the development of rectal microbicides (RMs) [9], which are topical agents designed to reduce HIV acquisition risk during receptive anal intercourse (RAI)​.

Rectal douching is a common practice among YMSM prior to engaging in RAI [10–13], with studies estimating between 50% and 90% of MSM engage in this behavior [14]​. Given its widespread use, researchers have explored the feasibility of integrating microbicides into rectal douches as a potential HIV prevention strategy [12, 13, 15–18]​. Prior research has demonstrated that individuals who regularly engage in rectal douching exhibit a greater willingness to use microbicide-containing douches compared to those who do not engage in this practice [10, 12, 13]​. This highlights the potential for a rectal microbicide douche to serve as an alternative to daily oral PrEP, particularly among populations with suboptimal PrEP adherence​.

The field of rectal microbicide research has employed several conceptual frameworks to assess product acceptability, recognizing that acceptability is a multidimensional construct encompassing perceptions of ease of use, comfort, perceived efficacy, and likelihood of future adoption [19–26]​. Mensch et al.’s framework [27] for microbicide acceptability posits that three key factors influence user willingness to adopt a product: [1] product characteristics (e.g., formulation, texture, perceived side effects) [2], delivery mechanism (e.g., mode of administration, ease of use), and [3] dosing regimen (e.g., frequency, timing relative to sexual activity)​. Similarly, Morrow and Ruiz’s acceptability model [28] highlights the role of behavioral congruence, emphasizing that prevention methods must align with individuals’ existing sexual and hygiene routines to maximize uptake​. These frameworks have been instrumental in guiding microbicide development and have informed the present study’s approach to evaluating the acceptability of a rectal microbicide douche among YMSM.

Understanding YMSM’s perspectives on rectal microbicide acceptability is critical for advancing the development of novel, behaviorally congruent HIV prevention products. While several prior studies have assessed the hypothetical acceptability of rectal microbicides among MSM populations, few have evaluated real-world acceptability following actual product use—particularly among YMSM, a group disproportionately affected by HIV and underrepresented in PrEP innovation research. ATN-DREAM (ATN 163) was designed to assess the real-world acceptability of a rectal microbicide douche candidate among YMSM—a key population for whom PrEP adherence remains suboptimal. ATN 163 assessed the safety, pharmacokinetics, and tolerability of a single 600 mg dose of tenofovir-based (TFV) rectal microbicide douche delivered in 125 mL of 0.45% sodium chloride (half-normal saline). As part of the study, we evaluated the acceptability of this novel rectal microbicide douche among YMSM who participated in this Phase I, open-label, single-arm clinical trial. The present analysis focuses on behavioral measures of acceptability, including participants’ immediate post-dosing experiences, long-term impressions, and willingness to use the product in the future. By focusing on real-time feedback after product administration, our findings provide unique insights into the behavioral congruence, perceived usability, and future uptake potential of rectal microbicides within this priority population, which may inform future iterations of rectal microbicides and contribute to ongoing efforts to expand HIV prevention options for YMSM.

## Methods

### Study Design

This study focuses on the acceptability of a tenofovir (TFV) rectal microbicide douche among adolescent and YMSM who participated in a Phase I, open-label, single-arm clinical trial designed to assess the safety, pharmacokinetics (PK), pharmacodynamics (PD), tolerability of a single 600 mg dose of TFV delivered in 125 mL of 0.45% sodium chloride (half-normal saline) and randomized to one of four post-dosing biopsy time points: 1 h, 6 h, 24 h, or 72 h. The study population included HIV-negative YMSM aged 18–24 who were sexually active, had engaged in RAI in the previous six months, and participated in the clinical trial. Participants were assigned male at birth, identified as male, were HIV-negative at screening, and had the ability to provide informed consent.

The product was prefilled by study staff and administered by a clinician as a single dose at the clinic. No at-home or multiple dosing was involved in this Phase I study. Participants completed a post-dosing survey following the administration of the rectal douche at the dosing visit and an exit survey focused on participants’ long-term impressions of the product’s acceptability and their willingness to use it in the future. In-depth interviews (IDIs) were conducted to gather detailed insights into their experiences using the rectal douche within a week after product use. Participants were instructed that the product was intended for use prior to RAI, aligning with typical douching behavior, and were asked to consider using the douche in that context when reflecting on acceptability and future use.

All participants were recruited from the Baltimore metropolitan area and underwent study procedures at Johns Hopkins University. Participants were compensated up to $400 for completing the study visit procedures ($150 for screening visits, $100 for completing the dosing visit, and $150 for completion of the final visit), per IRB-approved guidelines. Participants received an additional $100 for completing the in-depth interview. The trial was conducted in compliance with U.S. Department of Health and Human Services regulations and the Declaration of Helsinki. Institutional Review Board (IRB) approval was obtained. Full trial results related to PK/PD are published elsewhere (NCT04686279).

## Behavioral Survey

Following the administration of the rectal douche at the dosing visit, participants completed a post-dosing survey. This survey was designed to capture participants’ immediate reactions to using the product, with particular attention to its acceptability and tolerability. Questions covered potential side effects such as discomfort, bloating, and leakage, as well as overall satisfaction with the product’s administration (see Table 1).

**Table 1:** Acceptability indicators completed by YMSM following their TFV rectal douche dosing (*n* = 8)

Survey Question	Response Distribution
Would you consider using the rectal microbicide douche if it protected against HIV?	Highly Acceptable (7; 87.5%), Somewhat Acceptable (1; 12.5%), Somewhat Unacceptable (0; 0%), Completely Unacceptable (0; 0%)
Overall, how much did you like the douche?	Liked a little (5; 62.5%), Neither liked nor disliked (3; 37.5%), Disliked a little (0; 0%), Disliked very much (0; 0%)
How much did you like how the douche felt inside your rectum?	Liked a little (3; 37.5%), Neither liked nor disliked (3; 37.5%), Disliked a little (2; 25%), Disliked very much (0; 0%)
Did you experience bloating?	None (8; 100%), Some (0; 0%), A lot (0; 0%)
Did you experience gassiness?	None (5; 62.5%), Some (3; 37.5%), A lot (0; 0%)
Did you experience stomach cramps?	None (6; 75%), Some (1; 12.5%), A lot (1; 12.5%)
Did you experience pain?	None (5; 62.5%), Some (2; 25%), A lot (1; 12.5%)
Did you experience leakage/soiling?	None (6; 75%), Some (1; 12.5%), A lot (1; 12.5%)
Did you experience diarrhea?	None (7; 87.5%), Some (1; 12.5%), A lot (0; 0%)
Did the study douche feel cold?	Yes (5; 62.5%), No (3; 37.5%)
Would you be willing to use the product before every RAI?	Very Likely (6; 75%), Likely (2; 25%), Unlikely (0; 0%), Very Unlikely (0; 0%)
Would you be willing to use the product after every RAI?	Very Likely (3; 37.5%), Likely (3; 37.5%), Unlikely (2; 25%), Very Unlikely (0; 0%)
Would you use the product before every RAI with a one-night stand?	Very Likely (8; 100%), Likely (0; 0%), Unlikely (0; 0%), Very Unlikely (0; 0%)
Would you use the product before every RAI with a regular partner?	Likely (4; 50%), Very Likely (1; 12.5%), Unlikely (2; 25%), Very Unlikely (1; 12.5%)
Would you use the product before every RAI with other male partners?	Very Likely (8; 100%), Likely (0; 0%), Unlikely (0; 0%), Very Unlikely (0; 0%)

At the termination visit, participants completed an exit survey that gathered comprehensive feedback on their experiences throughout the study (see Table 2). The exit survey focused on participants’ long-term impressions of the product’s acceptability and their willingness to use it in the future. The survey explored how participants compared the rectal microbicide douche to other HIV prevention methods, such as condoms and daily oral PrEP, providing insights into their preferences for future use​.

**Table 2:** Acceptability indicators at termination visit (*n* = 8)

Survey Question	Response Distribution (*n*;%)
How likely would you be to use the rectal douche before receptive anal sex if it provided some protection against HIV?	Very Likely (6; 75%), Likely (2; 25%), Unlikely (0; 0%), Very Unlikely (0; 0%)
Would it be easy to use the PrEP douche?	Strongly Agree (7; 87.5%), Agree (1; 12.5%), Disagree (0; 0%), Strongly Disagree (0; 0%)
Would you have concerns about side effects of the PrEP douche?	Strongly Agree (1; 12.5%), Agree (1; 12.5%), Disagree (5; 62.5%), Strongly Disagree (1; 12.5%)
Would using the PrEP douche impact your daily routine?	Strongly Agree (5; 62.5%), Agree (3; 37.5%), Disagree (0; 0%), Strongly Disagree (0; 0%)
Would the PrEP douche be uncomfortable to use?	Strongly Agree (4; 50%), Agree (2; 25%), Disagree (0; 0%), Strongly Disagree (2; 25%)
Would you have to worry about remembering to use the PrEP douche?	Strongly Agree (6; 75%), Agree (2; 25%), Disagree (0; 0%), Strongly Disagree (0; 0%)
Would you have to worry about the PrEP douche being stolen?	Strongly Agree (6; 75%), Agree (2; 25%), Disagree (0; 0%), Strongly Disagree (0; 0%)
Would you have to worry about someone knowing you use the PrEP douche?	Strongly Agree (4; 50%), Agree (3; 37.5%), Disagree (1; 12.5%), Strongly Disagree (0; 0%)
Would you have to worry about how alcohol or drug use affects PrEP douche use?	Strongly Agree (3; 37.5%), Agree (3; 37.5%), Disagree (1; 12.5%), Strongly Disagree (1; 12.5%)
Would using the PrEP douche make your sex life more pleasurable?	Strongly Agree (4; 50%), Agree (4; 50%), Disagree (0; 0%), Strongly Disagree (0; 0%)
Would you be able to stop using the PrEP douche whenever you wanted?	Strongly Agree (5; 62.5%), Agree (2; 25%), Disagree (1; 12.5%), Strongly Disagree (0; 0%)
Would you feel protected against HIV using the PrEP douche?	Strongly Agree (6; 75%), Agree (2; 25%), Disagree (0; 0%), Strongly Disagree (0; 0%)
Which would you prefer to use as the receptive partner: the study douche, male condom, or both?	Both equally (5; 62.5%), Douche (3; 37.5%), Male condom (0; 0%), Neither (0; 0%)
Which would you prefer to use as the receptive partner: the study douche, daily oral PrEP, or both?	Both equally (2; 25%), Douche (6; 75%), Oral PrEP (0; 0%), Neither (0; 0%)

## In-Depth Interviews (IDIs)

Following the completion of clinical trial procedures, in-depth interviews (IDIs) were conducted to gather detailed insights into their experiences using the rectal douche. All qualitative interviews were conducted virtually, lasting 45–60 minutes, and followed a semi-structured interview guide designed to explore key dimensions of product acceptability as outlined in Mensch’s framework [27]: product characteristics, delivery mechanism, and dosing regimen. Participants were also invited to share their motivations for trial participation, their experiences with the product, and their thoughts on future use. Example questions included: ‘What were your overall experiences with the study rectal douche?‘, ‘If you could change the rectal douche in any way, what would make it better?’, and ‘Thinking about your own life experiences, what issues would prevent you from using the douche before anal sex?’. Interviews were audio-recorded, transcribed verbatim, and de-identified to protect participant confidentiality.

### Data Analysis

We employed a convergent mixed methods approach [29] to comprehensively assess the acceptability of the rectal microbicide douche among YMSM. Qualitative and quantitative data were analyzed separately and then integrated to provide a nuanced understanding of facilitators and barriers to adoption. Although the sample size was small, we employed a parallel design that integrated qualitative and quantitative data to enhance interpretive depth.

A thematic analysis approach was used to systematically identify patterns related to the acceptability of the rectal microbicide douche [30]. An a priori codebook was developed based on Mensch’s framework, incorporating categories related to ease of use, tolerability, integration into existing sexual health practices, perceived advantages over other HIV prevention methods, and potential barriers to adoption. The data were coded using NVivo 13 qualitative analysis software, with all interviews double-coded by three independent team members (JW, TB, LL). Discrepancies in coding were reviewed through consensus discussions to ensure reliability and interpretive validity. Thematic saturation was achieved by the seventh interview, with no new themes emerging in the final transcript.

Quantitative data from post-dosing surveys were analyzed using descriptive statistics to assess the frequency of key acceptability indicators, including perceived ease of use, willingness to use the product in different sexual contexts, and comparisons to other HIV prevention methods. Univariate statistics were conducted using SPSS 20 software.

We then triangulated the qualitative and quantitative findings, whereby we mapped the qualitative themes onto quantitative survey responses to identify converging, diverging, or complementary insights. This mixed-methods approach allowed for a comprehensive assessment of acceptability, integrating both qualitative insights and quantitative patterns to identify key facilitators and barriers to the adoption of the rectal microbicide douche among YMSM.

## Results

The study sample (*N* = 8) consisted of young adults with a mean age of 20.9 years (SD = 2.1; see Table 3). The majority identified as White, Non-Hispanic (*n* = 5; 62.5%), while the remaining participants were Black, Non-Hispanic (*n* = 3; 37.5%). Most participants identified as gay (*n* = 6; 75.0%), with 2 (25.0%) identifying as bisexual. Half (*n* = 4; 50.0%) were enrolled in school full-time, 1 (12.5%) attending part-time, and 2 (25.0%) were not enrolled.

**Table 3:** Demographic characteristics of YMSM who participated in the ATN-DREAM phase I dosing trial (*n* = 8)

Variable	*N* = 8
Age in years (mean ± SD)	20.9 ± 2.1
Race/Ethnicity	
White, Non-Hispanic	5 (62.5%)
Black, Non-Hispanic	3 (37.5%)
Sexual Orientation (%)	
Gay	6 (75.0%)
Bisexual	2 (25.0%)
Enrolled in school (%)	
Yes, Full-time	4 (50.0%)
Yes, Part-time	1 (12.5%)
No	2 (25.0%)
Missing/Refuse to Answer	1 (12.5%)
Highest education attained/currently pursuing (%)	
9th -12th grade	1 (12.5%)
GED program	1 (12.5%)
Four-year college	3 (37.5%)
Vocational school	2 (25.0%)
Missing/Refuse to Answer	1 (12.5%)
Sexual behavior in the past 3 months (%)	
No sexual partners	1 (12.5%)
1 + partners, no recent receptive anal intercourse (RAI)	2 (25.0%)
1 + partners, had recent RAI	5 (62.5%)
Ever used PrEP (%)	
No	8 (100.0%)
Yes	0 (0.0%)
Previous douching experience and frequency (%)	
Yes, always	3 (37.5%)
Yes, sometimes	2 (25.0%)
Yes, rarely	1 (12.5%)
No	2 (25.0%)
Reasons for douching (%)^‡^	
For general hygiene	3 (37.5%)
In preparation for sex	5 (62.5%)
For pleasure	3 (37.5%)
When constipated	1 (12.5%)

^‡^ Participants could select multiple responses; percentages sum to more than 100%

When asked about their sexual behavior in the prior three months, 5 (62.5%) had at least one partner and engaged in recent RAI. None had previously used PrEP. Douching behaviors were common, with 3 (37.5%) always douching, 2 (25.0%) sometimes douching, and 1 (12.5%) rarely doing so; the remaining 2 (25.0%) participants had never douched. Primary reasons for douching included preparation for sex (*n* = 5; 62.5%), general hygiene (*n* = 3; 37.5%), and pleasure (*n* = 3; 37.5%); 1 participant (12.5%) reported douching when feeling constipated.

### Product Characteristics

Participants reported high acceptability of the rectal microbicide douche, highlighting its ease of use, familiarity, and integration into existing sexual health routines. A majority (5 out of 8) described the product as intuitive, with one participant stating, “The study product was wonderful because it felt so easy and effortless to use… it was easy to apply, it was easy to insert, and it was easy to use.” (19-year-old, Black bisexual man). The design and format of the douche were also well received, particularly by those with prior douching experience, as it resembled commercial douches. Another participant emphasized that its packaging made it accessible: “Somebody who’s used a douche in the past will know how to use this. It’s very accessible for somebody who already understands the process of anal douching.” (21-year-old, White gay man).

Convenience was a critical factor influencing acceptability, specifically when compared to daily oral PrEP. Participants valued the episodic use of the rectal microbicide, with 7 (87.5%) stating they would consider using it if proven effective for HIV prevention. When assessing overall satisfaction, 5 (62.5%) indicated they liked the product a little, while 3 (37.5%) remained neutral. None of the participants disliked the product. Similarly, when asked about how the product felt inside the rectum, 3 (37.5%) reported that they liked it a little, while another 3 (37.5%) were neutral, and 2 (25%) disliked it a little.

## Tolerability and Side Effects

Side effects were minimal, with most participants reporting no adverse symptoms. While gassiness was reported by 3 (37.5%) participants, none reported bloating. Similarly, 1 (12.5%) participant experienced some cramps and another participant (12.5%) experienced a lot of cramping. Regarding pain, 5 (62.5%) participants experienced none, while 2 (25%) reported some pain, and 1 (12.5%) described a lot of pain. Similar trends were observed for leakage/soiling, with 1 (12.5%) participant experiencing some and another (12.5%) experiencing a lot of leakage/soiling. Diarrhea was rare, with one participant (12.5%) experiencing some diarrhea. The most frequently reported sensory reaction was coldness, with 5 (62.5%) participants noting that the product felt cold, while 3 (37.5%) did not perceive this sensation.

## Delivery Mechanism

Participants overwhelmingly viewed the rectal microbicide douche as a behaviorally congruent HIV prevention method, particularly for individuals who routinely douche before receptive anal intercourse (RAI). Nearly all (7 out of 8) reported prior douching experience, reinforcing that integrating an HIV prevention product into this established routine would be feasible. One participant noted, “I douche before receptive anal sex every time, so adding an extra layer of protection on top of that makes a lot of sense.” (18-year-old, White gay man).

Participants also positively commented on the product’s design, particularly the lubricated nozzle, which facilitated ease of insertion. However, some aspects of the design were less well received. Four participants (50%) expressed concerns about the thickness of the plastic, which made squeezing the bottle difficult, with one stating, “The nozzle wasn’t as smooth, and the plastic could have been better—it was a little hard to get every last drop out.” (24-year-old, White gay man). The single-use nature of the product also raised concerns, particularly among those who preferred reusable options for sustainability. One participant remarked, “I[‘d] like to be a little bit more sustainable… I want to make it as eco-friendly as possible. So, the disposable nature of it wasn’t as enticing.” (21-year-old, White gay man).

Despite these minor concerns, most participants (*n* = 5; 62.5%) reported no hesitations about future use and noted that self-administration would enhance usability.

### Dosing Regimen and Future Use Intentions

As shown in Table 2, acceptability was high. Seven (87.5%) participants stating they would consider using a rectal microbicide douche if it were proven effective in preventing HIV. Six (75%) participants indicated they would be likely to use it before every instance of RAI. Participants identified key populations that would benefit most from the rectal douche for HIV prevention, with MSM communities frequently mentioned as the target population who would benefit most from a rectal microbicide douche. One participant noted, “I think a lot of that demographic already uses [an] anal douche, so just making a super douche would be right up their alley—literally.” (21-year-old, White, gay man). Additionally, younger adults, particularly those aged 18–30, were identified as a priority group, as they may engage in higher-risk sexual behaviors. One participant described this demographic as particularly well-suited for integrating the product into their routine, stating, “Younger gay men usually take more risks in terms of sex, so having more protection integrated into a routine would just be better for them.” (18-year-old, White gay man).

The episodic nature of the rectal microbicide douche was perceived as an advantage. All participants agreed that they would strongly consider using the product in higher-risk sexual encounters. One participant summarized this sentiment: “I just really thought that having an HIV douche as another way of preventing HIV makes sense because a lot of gay men already douche. Having an extra layer of protection on top of that would be good—especially in situations where they’re more at risk, like one-night stands.” (18-year-old, White gay man).

Participants endorsed the use of a rectal microbicide douche before engaging in receptive anal intercourse with casual partners or when a partner’s HIV status was unknown: “[I would use it] Before maybe a hookup, or just before having sex with your partner, or sex with anyone” (19-year-old, Black bisexual man) and in “high-risk situations where it’s unknown or you’re not fully trusting of your partner’s status”. (23-year-old White gay man). These qualitative insights coincide with participants’ survey responses: willingness to use a rectal microbicide douche was highest for casual encounters, with 100% reporting they would be very likely to use it before a one-night stand. However, willingness decreased slightly for regular partners: 4 (50%) were likely, 1 (12.5%) very likely, 2 (25%) unlikely, and 1 (12.5%) very unlikely to use it.

When asked to compare the rectal microbicide douche with other HIV prevention methods (see Table 2), participants most frequently referenced daily oral PrEP and condoms. Several participants expressed a preference for the rectal douche over daily oral PrEP due to its event-driven nature and perceived convenience. These preferences were reflected in survey responses: when asked which prevention method they would prefer to use as the receptive partner, 6 (75.0%) chose the rectal douche over daily oral PrEP, while 2 (25.0%) rated both equally; none selected oral PrEP alone. Similarly, when comparing the rectal douche to condoms, 3 (37.5%) preferred the douche, and 5 (62.5%) rated both equally; no participants preferred condoms alone or rejected both options. Although participants were not explicitly asked about other PrEP modalities such as 2-1-1 (event-driven) PrEP or long-acting injectables, these alternatives were not mentioned spontaneously during interviews. The absence of such comparisons suggests a potential gap in participant awareness or salience of these options at the time of the study.

### Future Product Refinements and Recommendations

Participants identified several areas for refinement to enhance the usability, appeal, and adoption of the rectal douche for HIV prevention. Key recommendations focused on improving product design, addressing comfort and stigma-related concerns, ensuring affordability and privacy, and expanding educational outreach to support long-term adherence.

Participants emphasized the importance of usability and comfort in the adoption of the rectal douche for HIV prevention. As one participant suggested, “If the bottle were easier to squeeze and maybe slightly warmed up before use, I think it would make the experience even better.” (23-year-old, White gay man). Additionally, participants underscored the need for a design that facilitates ease of use, particularly in spontaneous situations. Participants noted the product’s convenience as a factor supporting its adoption, with one stating, “It would be so easy and convenient just to stop by a drug store, pick it up on your way home or just to go out real quick for five minutes, come back home, use it, and then you’re ready to go.” Another participant described the product as “so easy to use…it’s really great…it’s worth it and it’s better than the average anal douche.” (19-year-old, Black bisexual man).

Three participants also remarked that the study product could help address PrEP-related stigma given its congruence with pre-RAS behaviors. As one participant elaborated, “[the study product has] potential to break through a little bit of this stigma there… In a theoretical world, I can see a situation where someone who may still be experiencing internalized homophobia and discomfort with their sexuality might be more comfortable utilizing this because it’s already looks like how they prepare for receptive anal sex versus having to add the extra steps of taking oral medication.” (21-year-old, White gay man).

Affordability was a significant factor influencing potential product adoption in the future. Two participants expressed concern that pricing could serve as a barrier, particularly for individuals without access to insurance coverage or healthcare programs. One participant noted, “If it’s too expensive, people just won’t use it, even if they want to.” (19-year-old, Black bisexual man). Cost was also reflected in participants’ trade-offs regarding the value-added of using the study product, highlighting the importance of designing a product that was reusable. As one participant mentioned: “I imagine there’s going to be a cost with it…so it will be easier just to use the reusable douche that I have now.”. (18-year-old, White gay man).

Privacy emerged as an additional consideration to product adoption. As one participant mentioned, “a lot of young people are having to live with their parents for longer…also think roommates can be a factor…so I think that can also play a factor and obviously changes your availability to a restroom or a bathroom, where you have the time to sit down for administer the product and let it sit for five or 10 minutes, or however long it has been shown to be effective, and allowing the tenofovir to enter the bloodstream.” (21-year-old, White gay man). Based on these insights, future iterations should address design considerations (e.g., coldness concerns; reusability), strategies to address cost barriers, and long-term adherence strategies to optimize user uptake.

## Discussion

In this study, we assessed YMSM’s acceptability of a tenofovir-based rectal microbicide douche using Mensch’s acceptability framework [27], which examines product characteristics, delivery mechanism, and dosing regimen. Our findings indicate that the rectal microbicide douche was highly acceptable due to its ease of use, alignment with existing douching behaviors, and episodic dosing schedule. These results contribute to the growing body of literature demonstrating that behaviorally congruent HIV prevention modalities have the potential to improve adherence and uptake among YMSM populations [2, 8, 19, 21]​.

Similar to previous research on rectal microbicide gels [22–24, 31–33], participants in our study reported high ease of use and integration into their sexual health routines​. Unlike microbicide gels, which have required the use of an applicator, the douche format was perceived as intuitive, particularly among individuals with prior douching experience. This supports findings from prior studies where microbicide acceptability was linked to the degree of behavioral familiarity with a given product [12, 13, 15, 19, 34]​. Participants highlighted the convenience of the rectal douche, particularly compared to daily oral PrEP, reinforcing previous findings that event-driven prevention strategies may be preferable for those who struggle with daily medication adherence​. Despite these positive perceptions, however, YMSM expressed some concerns about the product usability, which made administration challenging. Prior studies on microbicide gels have similarly noted that product viscosity and application mechanisms influence user experience and willingness to adopt these products long-term ​ [12, 13, 15, 19, 34]​. Addressing these usability concerns in future product development—such as incorporating a softer plastic material—may further enhance user acceptability. In addition to product characteristics, some participants raised concerns about privacy when living with others, as well as access and cost—echoing themes identified in broader PrEP research and policy.

In our study, participants indicated that they would find a rectal microbicide douche acceptable if it were proven effective, and reported that they would be likely to use it before every instance of RAI. The high acceptability of the rectal microbicide douche was largely attributed to its behavioral congruence with existing douching practices, aligning with previous research that emphasizes the importance of embedding prevention strategies within routine sexual health behaviors ​ [10, 12, 19, 21, 35]. Most participants in our study reported regular douching prior to RAI, suggesting that the rectal microbicide douche could be easily incorporated into their pre-sex routines. While the sample was too small for subgroup analysis, our findings suggest that those without prior rectal douching experience were still open to using the study product in the future. Moreover, the episodic nature of the rectal microbicide douche was viewed as an advantage, particularly for individuals who engage in casual sexual encounters or one-night stands. This finding aligns with research suggesting variability in PrEP willingness based on partner type [36–38].

Most participants in our study preferred the rectal microbicide douche over daily oral PrEP, citing its event-driven nature and alignment with existing sexual health routines as key advantages. This preference may reflect the fact that all participants were not currently using daily oral PrEP at the time of enrollment, suggesting that a rectal microbicide may be more desirable for YMSM who are unable or unwilling to use systemic PrEP modalities. When asked to compare prevention methods, one-third of participants preferred the rectal douche over condoms, while 62.5% rated both equally—highlighting its appeal even relative to other event-driven strategies. Taken together, these findings underscore the importance of offering an array of prevention choices to YMSM; that is, viewing event-driven products like a rectal microbicide douche as a complement to other modalities including systemic PrEP and condoms [7, 8, 20, 35]​ in order to provide YMSM with multiple methods of protection. Moreover, although participants strongly favored the rectal douche over daily oral PrEP, we did not include direct prompts regarding other emerging options such as 2-1-1 PrEP or long-acting injectable PrEP in our survey assessments or interview guides. Participants also did not discuss these in their conversations, highlighting the limited awareness of or access to these alternatives at the time of data collection. Future research should explore how rectal microbicides compare to other non-daily PrEP options to better understand how YMSM make decisions among available modalities and to inform more tailored, user-centered prevention strategies.

Previous research on rectal microbicide gels has shown that adherence may be lower than anticipated due to timing concerns, with some participants noting that stopping to apply a product before sex could be disruptive ​ [22–24, 39]. Although timing concerns were raised in our study, half of the participants reported that it would not be a barrier to use, as they had already planned for douching before RAI. These findings suggest that future rectal microbicide interventions should explore optimized formulations that minimize pre-sex preparation time. Research has also highlighted that cost concerns may impact willingness to adopt new HIV prevention technologies, particularly among young individuals with limited healthcare access [7, 8, 40]​. Some participants in our study expressed concerns about the privacy of douching, particularly for those who live with family or roommates. To enhance usability and uptake, future product development should focus on improving product design (e.g., softer squeeze bottles), providing reusable or refillable options, and developing educational campaigns to promote awareness. Prior studies on microbicide gels have emphasized the importance of addressing product usability concerns early in development, as these factors significantly influence long-term adherence ​ [27, 28, 38, 41].

This study contributes to the growing body of research on rectal microbicides by providing qualitative insights into product acceptability among YMSM. Using Mensch’s acceptability framework allowed for a comprehensive assessment of product characteristics, delivery mechanisms, and dosing preferences. Additionally, the mixed-methods approach combining qualitative interviews and quantitative survey data provided a holistic understanding of user experiences. However, several limitations should be noted. The sample size was small and geographically limited, potentially limiting generalizability. Additionally, participants were enrolled in a clinical trial setting, which may not fully reflect real-world usage patterns. However, this study represents a rare opportunity to assess acceptability following actual product use rather than hypothetical scenarios. This is a notable strength, as it provides more ecologically valid insights into user experience, including sensory reactions, perceived barriers, and the feasibility of incorporating such a product into real-life routines. Future studies should explore longitudinal acceptability and adherence patterns in diverse settings, including non-research environments.

## Conclusions

Findings from ATN DREAM show that a rectal microbicide douche was highly acceptable to YMSM, largely due to its ease of use, alignment with existing sexual health behaviors, and on-demand dosing. Unlike studies based on hypothetical scenarios, our results are grounded in participants’ actual experiences with the product—offering insight into its physical feel, privacy concerns, and how it compares to other HIV prevention options. This next to real-world perspective allows for a more accurate assessment of behavioral fit and highlights areas for improvement, including product design, cost, and accessibility. Participants especially valued the douche for higher-risk encounters and preferred it over daily oral PrEP. These findings support the potential of rectal microbicide douches as a behaviorally congruent HIV prevention strategy for populations with low PrEP adherence, and offer critical guidance for developing user-centered, multipurpose prevention technologies.
